# Analysis of chemical constituents in mainstream bidi smoke

**DOI:** 10.1186/s13065-019-0614-7

**Published:** 2019-07-22

**Authors:** Omobola Ajoke Oladipupo, Dibyendu Dutta, Ngee Sing Chong

**Affiliations:** 1 Department of Chemistry, Middle Tennessee State University, P.O. Box 68, Murfreesboro, TN 37132 USA; 2 Present Address: Eli Lilly and Company, Lilly Technology Center North, 1220 West Morris Street, Indianapolis, IN 46221 USA; 3 Department of Professional Science, Middle Tennessee State University, P.O. Box 83, Murfreesboro, TN 37132 USA

**Keywords:** Tobacco smoke, Bidi cigarettes, Size-fractionated analysis of particulate matter, Sioutas cascade impactor, FTIR, GC–MS analysis of bidi emissions

## Abstract

Bidi, an indigenous form of cigarette in South Asian countries, is popular because of its low cost and multi-flavored variants. Although recent studies have shown that bidi smokers suffer from various adverse health effects including cancer, research on bidi smoke composition and exposure levels is still very limited. In this research, the vapor and particulate phases of bidi were characterized using gas chromatography coupled with mass spectrometry (GC–MS) and Fourier-transform infrared spectrometry (FTIR). The amounts of nicotine, cotinine, indole, substituted phenols, substituted pyridines, and phytol found in different size fractions of the particulate matter collected using a cascade impactor were reported. Due to the low combustibility of the tendu leaf in bidi, a six-second puff interval was used to sample the smoke constituents for analysis. Significant levels of carbon monoxide, hydrogen cyanide, and hydrocarbons like ethylene, methane and 1, 3-butadiene were detected in the mainstream bidi smoke. In addition, 3-methylpyridine, cotinine, α-amyrin, and β-amyrin were also present at high levels in bidi smoke. Despite having less tobacco compared to conventional cigarette, bidi smokers are potentially exposed to significantly higher concentrations of nicotine due to the greater puffing frequency. The non-porous nature and higher moisture content of tendu leaf in bidis compared to cigarette wrapping paper led to higher levels of carbon monoxide and tar in bidi smoke compared to regular cigarette smoke. Results of this study indicate the presence of harmful and carcinogenic chemicals in the mainstream bidi smoke that could be harmful to human health.

## Introduction

Bidis are slim, cylindrical, hand-rolled cigarettes indigenous to South Asian countries [3]. Due to its relatively low cost (a pack containing 20 pieces of bidis in USA costs $4.00–$6.00, whereas in India it is $0.08–$0.17) [11], bidis are popular among low and middle-income countries, and get imported from India to more than 122 countries worldwide, including the USA [11]. A report from Centers for Disease Control and Prevention (CDC) stated that 0.8% of all middle school students and 0.9% of all high school students in the USA smoked bidi during the period of 2011–2014 [4]. Although the underdeveloped and developing countries account for about 80% of one billion global smoking population and six million premature deaths annually, research on the characterization of bidi smoke is limited [37]. The absence of active research focused on bidi smoke toxicity by health organizations in South Asian countries is potentially alarming considering bidi smoking is 7–8% more popular in these countries compared to conventional cigarette smoking [7]. While global in-depth exposure and toxicological studies of conventional cigarette smoke is readily available, research on bidi smoke is considerably less. The lack of an approved analytical protocol for testing bidi, similar to that of the ISO 3308 method for testing of cigarettes, have also limited bidi smoke research. Bidi smoking has been associated with many adverse health conditions including cancer and cardiopulmonary diseases [27]. Research from India have shown a direct correlation between bidi smoking and several diseases such as lung cancer and coronary heart diseases [40], head and neck cancers [30], oral cavity and pharyngeal cancers [14], and Aspergillus sensitization [1]. There is a greater prevalence among heavy bidi smokers who show low ventilatory capacity and cardiorespiratory symptoms including wheezing, coughing, dyspnea or shortness of breath, and chest pain compared to non-smokers. Consequently, there is a greater baseline respiratory morbidity and higher risks of mortality linked to heavy bidi smokers, especially among low socio-economic and elderly smokers [9]. Despite many reports on the harmful effects of bidi smoking, the current knowledge about the chemical constituents of the mainstream bidi smoke (smoke that goes through the length of the bidi before being inhaled) is minuscule.

Tobacco smoke from conventional cigarette is a highly complex and dynamic mixture of more than 6500 constituents [29] with 150 harmful or potentially harmful substances [10] and more than 50 known carcinogens [12]. Many of these chemicals such as polycyclic aromatic hydrocarbons (PAHs), volatile organic compounds (VOCs), phenols, tobacco-specific nitrosamines (TSNAs), aromatic amines and metals are harmful and have carcinogenic effects [19, 20, 23]. While the mainstream cigarette smoke is very complex, bidi smoke may have additional compounds due to the unconventional ingredients used in bidi manufacturing. Bidis are composed of approximately 0.2 g of dark, sun-dried and processed tobacco flakes wrapped in a tendu leaf (*Diospyros elanoxylon*). The length of bidis may vary from 4 to 8 cm and the tendu leaf contributes 60% of its weight whereas, in conventional cigarettes, approximately 90% of the weight is from tobacco [11]. While the amount of tobacco present in bidis is approximately 0.2 g/piece, the amount of nicotine in bidis is very high (21.1 mg nicotine/g tobacco filler) [17]. Bidis may be filtered or unfiltered and may also contain flavoring agents. Due to the use of the non-porous tendu leaf that prevents ventilation and the lack of a filter section in the bidis, the amount of tar and carbon monoxide delivery during bidi smoking is very high. Replacing the tendu leaf with a porous cigarette wrapping paper reduces the amount of tar delivery by 66% [21]. Other mainstream smoke constituents detected from a 76 mm unfiltered bidi are acetaldehyde (751 μg), hydrogen cyanide (903 μg), ammonia (284 μg), isoprene (533 μg), acrolein (67 μg), *o*-cresol (55.3 μg), *m*- and *p*-cresol (139 μg), phenol (249 μg), 2,4-dimethylphenol (27.6 μg), *p*-ethylphenol (41.6 μg), and PAHs (benz[a]anthracene 117 ng and benzo[a]pyrene 78 ng) [13]. Among TSNAs, the mainstream smoke of bidis was found to contain more N’-nitrosonornicotine (NNN) compared to 4-(methylnitrosamino)-1-(3-pyridyl)-1-butanone (NNK) [38].

Analysis of bidi smoke, however, has been challenging because of the high variability in their manufacturing process. Unlike the production of American or European cigarettes, bidi manufacturing is a cottage industry that is highly unregulated, with minimal automation and quality control [11]. In addition, the ISO 3308 conditions designed for reproducibility of results among different brands of conventional cigarettes are not applicable to the experimental investigation of bidi smoke. The recommended parameters of ISO 3308 are: (a) puff volume of 35 ± 0.5 mL; (b) puff duration of 2.0 ± 0.2 s; (c) puff frequency of one per 60 ± 1 s and; (d) butt length of 23 mm for non-filter cigarettes or the length of filter plus 3 mm of filter overwrap [8]. However, due to the low combustibility and nonporous nature of tendu leaves, stronger puffs with increased puff volume and frequency are needed to light a bidi and keep it from self-extinguishing [22, 39]. Therefore, it is not realistic to acquire bidi smoke data based on the ISO 3308 protocol of 35 mL puff volume and puff frequency of one to two puffs per minute since it may not reflect the extent of toxicant exposure when bidi smoke is inhaled.

In this study, we analyzed the chemicals present in the vapor and particulate phases of the mainstream smoke of different types of bidi (flavored and unflavored). In an earlier report, measurement of total particulate matter (TPM) using one puff per 15 s have demonstrated the presence and higher delivery of nicotine, tar and carbon monoxide [36]. Therefore, in this study, we employed a puff duration of 6 s with a 6-s puff interval to simulate the smoking conditions for bidi in order to analyze the chemical constituents in mainstream bidi smoke. The smaller molecular weight compounds in the bidi smoke were analyzed by using Fourier-transform infrared spectrometry (FTIR) with a long-pathlength gas cell. Compounds with molecular weight larger than 50 g/mole (i.e. more than four carbons or larger than butane) were analyzed by gas chromatography coupled with mass spectrometry (GC–MS). Due to the differences in the physical and chemical characteristics of the bidis, (i.e. different amounts and sizes of sun-dried tobacco materials wrapped in tendu leaf) and the modification of the puffing regimen for sample collection, results obtained in this study are not comparable to those obtained with conventional reference cigarettes such as 1R4F and 1R5F.

## Materials and methods

### Materials

Three different flavored (vanilla, strawberry and grape) White Rhino filtered bidi packets (20 pieces/pack) (Kretek International, Moorpark, California, USA) were purchased from a local store in Brentwood, Tennessee, USA. An unflavored Seyadu bidi packet was obtained from India. The average weight of a flavored bidi was 0.483 g with 0.20 g tobacco/bidi. All bidis were marked at 23 mm from its butt in order to stop the bidi smoke sampling at this mark.

### Methods

#### Sampling

The analysis of bidi smoke was based on a puff-by-puff sampling procedure with a puff duration of 6 s spaced 6 s apart. The puffing regimen was based on the observation that, when smoking was done for less than 5 s with more than 6 s interpuff duration, bidi would self-extinguish due to the non-porosity and moisture content of the tendu leaf. In fact, it was reported that bidi smokers smoke with higher puff intensity and shorter interpuff duration [22, 39]. For smoke sampling, bidis were ensured to be sufficiently dry and equilibrated to room temperature before the collection of emission samples. A dried bidi was fitted snugly into a Teflon tubing. The length of the tubing was minimized to avoid interferences of phthalate esters present in the polymeric tubing. All connections were wrapped with Teflon tapes to ensure airtightness. The Aircheck personal sampling pump (224-PCXR7, SKC Inc., Eighty-Four, Pennsylvania, USA) was used to sample bidi smoke through the ASSET-32 activated charcoal tubes (28301-U, Sigma-Aldrich, St. Louis, Missouri, USA). A Y-junction was connected to the bidi so that the smoke was simultaneously sampled into an ASSET-32 sorbent tube at a flowrate of 1.05 L/min and into a multipath gas cell (Infrared Analysis Inc. Anaheim, California, USA) with an optical pathlength of 2.4 m and an internal volume of 95 mL. The DryCal DC-Lite primary flowmeter (Bios International, Pompton Plains, New Jersey, USA) was used to calibrate the required flowrates to the gas cell prior to each sampling event. For analysis of the mainstream smoke, an average of three puffs per bidi was used instead of more puffs to avoid instrumental detection saturation.

Analytes from the sorbent tubes were extracted with 1.5 mL methanol (GC–MS grade, Burdick and Jackson, Muskegon, Michigan, USA) and petroleum ether (OPTIMA grade, Fisher Scientific, Fair Lawn, NJ, USA) for GC–MS analysis. The sampling flowrate and duration of the ASSET-32 sorbent tubes without breakthrough occurrence were verified by placing two sorbent tubes in a series, followed by GC–MS analysis of the extracts from each tube, to ensure that the extract from the second tube did not show the presence of the compounds detected on the first tube.

A Sioutas cascade impactor (Catalog No. 225-370, SKC Inc., Eighty-Four, PA) was used as per the manufacturer’s instructions for sampling bidi smoke particulate phase onto filter pads. First, a sampling flowrate of 9.0 L/min was adjusted (flowrate recommended for air sampling with Sioutas cascade impactor) with the unburned bidi attached to the inlet of the impactor. During sampling, the flowmeter was removed and the outlet of the impactor was directly attached to the pump. All connections were tightly wrapped with Teflon tape. The bidi was then lit with a match stick and allowed to burn till the 23-mm mark of the bidi cigarette. At the sampling flowrate of 9.0 L/min, the smoke particles traversed the impactor such that the particles of different size fractions were collected successively on 0.50 μm PTFE (Teflon) filters of 25 mm diameter with a PTFE support (Catalog No. 225-1708, SKC Inc., Eighty-Four, Pennsylvania, USA) in the following order: Filter A (> 2.5 μm), Filter B (1.0–2.5 μm), Filter C (0.5–1.0 μm), and Filter D (0.25–0.5 μm). Particulate samples for the size fraction of < 0.25 μm were collected onto a 2.0 μm PTFE filter of 37 mm diameter (225–1709, SKC Inc., Eighty-Four, Pennsylvania) attached to the after-filter (Filter L). After sampling, the impactor was kept idle for 10 min and then disassembled in a dust-free environment. Analytes from the filters were extracted with 5.0 mL methylene chloride (HPLC grade, Fisher Scientific, Fair Lawn, New Jersey, USA) for 30 min in an ultrasonic bath (B22-4, Branson Company, Shelton, Connecticut, USA). Extracted samples were crimped in 2 mL amber vials (5181–3376, Agilent, Palo Alto, California, USA) and stored in the refrigerator prior to GC–MS analysis.

#### Smoke analysis

A Nicolet Magna 550 FTIR spectrometer (Madison, Wisconsin, USA) and a 2.4-m gas cell (Infrared Analysis Inc., Anaheim, California, USA) were used for analysis of bidi smoke. The infrared spectra were recorded using a Mercury Cadmium Telluride (MCT/A) detector at a resolution of 0.5 cm^−1^ with no zero filling and Happ-Genzel apodization. The gas cell was evacuated prior to the acquisition of the background FTIR spectra. The total spectral acquisition time for co-adding 100 scans was 7 min. The infrared spectra of bidi smoke were analyzed and compared to the online database of standard infrared spectra available at the website of Pacific Northwest National Laboratory. A 0.5 μm filter (SS-4F-VCR-7, Swagelok, Memphis, Tennessee, USA) was used to trap particulate matter in the bidi smoke prior to passing into the gas cell for FTIR analysis. The filter helped avoid the contamination of the gas cell and minimized the need for frequent cleaning of the gas mirrors.

An Agilent 6890 N gas chromatograph (GC) interfaced to an Agilent 5973 mass spectrometer (MS) was used for GC–MS analysis. The chromatographic separation was carried out using a Restek MXT-1 (Bellefonte, PA, USA) column (60 m × 0.25 mm i.d. and a film thickness of 0.50 μm) with 100% polydimethylsiloxane stationary phase. The oven was initially held at 50 °C for 3.00 min. The column was then heated at 8 °C/min to 170 °C and held for 1 min. The second temperature ramp was set at 15 °C/min and held at 220 °C for 10 min, and finally the temperature was increased to 320 °C at a temperature gradient of 20 °C/min with a final hold time of 6 min. Inlet temperature was kept at 250 °C and the helium carrier gas flow was maintained at a constant rate of 0.90 mL/min. The MS was operated in full scan and electronic impact ionization mode at 70 eV as well as a mass scan range of 35–550 amu. The GC–MS interface temperature was kept at 280 °C along with the ion source temperature of 300 °C.

For the quantitative determination of the major components of bidi smoke, standards based on high purity (> 99%) compounds of nicotine, cotinine, styrene, 2-methyl pyridine, indole, furfural, phenol, 2-methylphenol, 4-methylphenol, and 2-methoxyphenol were used to prepare calibration plots. For the identification of other compounds present in the bidi smoke, the library search and spectral matching of the mass spectra of compounds separated by GC were conducted using the National Institute of Standards and Technology (NIST) MS database. When similar match indices of different compounds were observed for GC–MS peaks, the observed retention times are plotted against NIST retention indices of possible matches to evaluate the degree of fit on the linear regression plot in order to confirm the identity of the compounds. For statistical analysis, sampling was done in triplicate and analyzed using Microsoft Excel. The concentration data of smoke constituents were reported as mean ± 1 standard deviation (SD).

## Results

### FTIR analysis

**Fig. 1:**
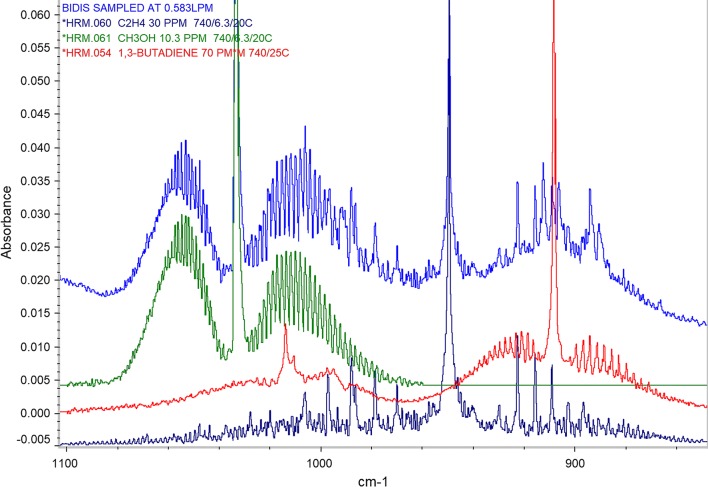
Comparison of the FTIR spectrum of bidi smoke collected at 0.583 L per minute and the reference spectra of ethylene, methanol and 1,3-butadiene

Figure 1 shows the infrared spectrum of unflavored Seyadu bidi mainstream smoke collected at 0.583 L per minute. The overlay of reference infrared spectra of methanol, ethylene and 1, 3-butadiene onto the spectrum of bidi smoke sample clearly shows the distinct presence of these compounds in bidi mainstream smoke. The FTIR spectrometer resolution of 0.5 cm^−1^ allows the identification of the smoke toxicants based on their characteristic infrared absorption signals with a peak width at half maximum of less than 2 cm^−1^. Small molecules including methanol, ethylene and 1, 3-butadiene along with methane, formaldehyde, hydrogen cyanide, carbon dioxide, and carbon monoxide have narrow peak widths and can be readily quantified using reference spectra of gas standards. The concentrations of some of these compounds are shown in Table 1. The concentrations of carbon monoxide and hydrogen cyanide obtained in bidi smoke using our sampling technique were 4.95 mg/bidi, and (310.8 μg/bidi), respectively.

**Table 1:** Concentrations (ppm) of highly volatile compounds detected in the mainstream bidi smoke by FTIR spectrometry at different sampling flowrates

Compound	Flowrate (L/min)			
	0.56	0.96	1.25	1.54
Carbon monoxide	4630 ± 604	3910 ± 907	2460 ± 633	2310 ± 570
Carbon dioxide	56,970 ± 7635	38,360 ± 4671	28,240 ± 5654	30,340 ± 840
Methanol	5140 ± 62	3330 ± 11,070	2490 ± 296	2860 ± 184
Methane	1930 ± 176	1360 ± 288	981 ± 73	980 ± 122
Ethylene	549 ± 99	585 ± 112	254 ± 36	234 ± 61
Ethane	1186 ± 73	680 ± 135	640 ± 222	498 ± 153

The data are reported as “mean ± 1 standard deviation” based on triplicate measurements

### Bidi smoke characterization by GC–MS analysis of ASSET-32 sorbent tube extracts

**Fig. 2:**
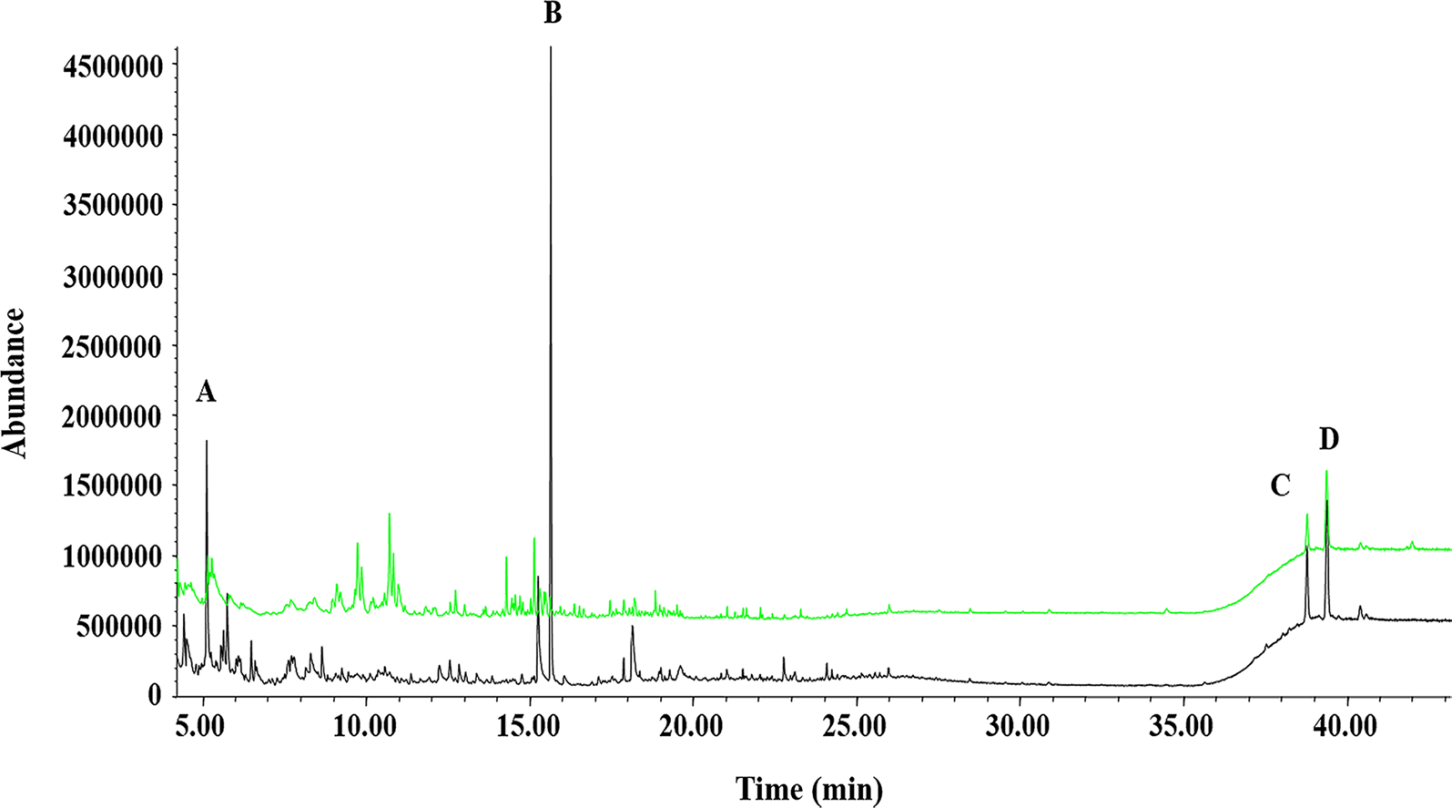
Overlaid GC–MS chromatogram of bidi smoke sampled onto ASSET-32 activated charcoal sorbent tubes and extracted with petroleum ether (green) or methanol (black). A Furfural, B nicotine, C alpha-amyrin and, D beta-amyrin

Constituents of bidi smoke samples were extracted from ASSET-32 tubes with petroleum ether and methanol, and identified using GC–MS. The compounds extracted by petroleum ether were relatively non-polar compounds such as 4-methyl octane, 1, 2-dimethylbenzene, 4-methyl undecane, hexadecane, and 1-docosene whereas polar compounds like alkylphenols and alkyl-substituted pyridines were found in the methanol extract (Table 2). Furfural, nicotine, α-amyrin, and α-amyrin were observed in the extracts of both methanol and petroleum ether. The GC–MS signals of these compounds had higher intensities and greater mass spectral match indices compared to those for petroleum ether extract (Table 2 and Fig. 2).

**Table 2:** Bidi smoke compounds identified by GC–MS analysis of petroleum ether and methanol extracts of Asset-32 sorbent tubes

Petroleum ether		Methanol	
Compound	Retention time (min)	Compound	Retention time (min)
4-Methyloctane	5.13	2-Methylpyridine	4.87
1,2-Dimethylbenzene	5.26	2-Furanmethanol	5.54
Styrene	5.83	3-Methylpyridine	5.62
Dodecane	9.84	2-Methyl-2-cyclopenten-1-one	6.46
4-Methylundecane	10.97	Phenol	8.29
Tetradecane	12.71	Limonene	9.06
Hexadecane	14.28	2-Methylphenol	9.89
Octadecane	17.63	2-Methoxyphenol	10.30
1-Docosene	24.67	4-Methylphenol	10.35
Heptacosane	28.44	Cotinine	21.03

Table 3 shows GC–MS analysis of bidi smoke sampled using ASSET-32 sorbent tubes that were extracted with methanol subsequently. Furfural, 2-oxo-3-cyclopentene-1-acetaldehyde, 2,6-dimethoxy-4-(2-propenyl) phenol, nicotine, and 3-(1-methyl-1*H*-pyrrole-2-yl)pyridine (nicotyrine) were present at high levels in bidi smoke. A GC–MS peak at the retention time of 12.9 min was identified as 2-ethyl-3-hydroxy-4*H*-pyran-4-one in the bidi smoke. In addition, the terpenoid aroma compound, d-limonene or 1-methyl-4-(1-methylethenyl)-cyclohexene, and pentacyclic triterpenes, α-amyrin and β-amyrin were also present at high levels in bidi smoke. Besides nicotine, bidi smoke was also found to contain relatively high levels of 3-methylpyridine, cotinine, and other polar compounds including alkyl phenols, furfural, and 2-furanmethanol.

**Table 3:** Concentrations of mainstream bidi smoke compounds detected by GC–MS analysis following methanol extraction of Asset-32 sorbent tubes

Compound	Concentration (μg/puff)	RSD^a^
3-Methylpyridine	39.0 ± 2.6	6.8
Styrene	7.2 ± 1.2	16.6
Cotinine	10.0 ± 3.6	36.4
Nicotine	497 ± 188	37.8
Furfural	1.8 ± 0.2	11.4
2-Furanmethanol	13.5 ± 3.0	22.4
Phenol	38.5 ± 1.1	6.8
2-Methylphenol	5.4 ± 0.9	16.5
4-Methylphenol	15.1 ± 1.4	9.5
2-Methoxyphenol	17.1 ± 0.9	5.3
2-Methoxy-4-methylphenol	3.4 ± 0.02	0.5

The concentration values are reported as “mean ± 1 standard deviation” for triplicate measurements

^a^RSD: Relative standard deviation

### GC–MS analysis of fractionated particulate matter in bidi smoke

Nicotine, observed in all size fractions, was the major component in the particulate phase of bidi smoke. The respective amounts of nicotine found in grape and vanilla bidis were 0.39 mg and 0.66 mg in Filter A (> 2.50 μm) and 18.21 mg and 20.20 mg in Filter L (< 0.25 μm) of the Sioutas cascade impactor, with Filter L containing the highest (80%) proportion of nicotine (Table 4). Other identified compounds in the particulate phase include 4-methylphenol, indole, 2-methoxy-4-vinylphenol, tetracosane, nonacosane, vitamin E, campesterol, α-amyrin and β-amyrin. The levels of 2-methylphenol, phytol, 2,3-dipyridyl, 3-(3,4-dihydro-2*H*-pyrrol-5-yl)pyridine or myosmine, 3-methyl-1*H*-indole, 4-methylphenol, cotinine, vitamin E, as well as α-amyrin and β-amyrin were the highest in the Filter L (< 0.25 μm) (Table 5). Compounds detected only in Filter L, the filter with smallest pore size, include styrene, 3-pyridinol, benzyl nitrile, squalene, 1, 2-benzenediol, 2-furanmethanol, maltol, 2-methyl-2-cyclopenten-1-one, megastigmatrieneone, campesterol, 22, 23-dihydrostigmasterol and naphthalene.

**Table 4:** Concentrations of nicotine (mg/bidi) for different size fractions of the particulate phase of bidi smoke as measured by GC–MS analysis

Bidi/cigarette	Filter				
	A (> 2.50 μm)	B (1.0–2.50 μm)	C (0.50–1.0 μm)	D (0.25–0.50 μm)	L (< 0.25 μm)
Grape bidi	0.39 ± 0.005	0.17 ± 0.002	0.16 ± 0.001	0.65 ± 0.006	18.21 ± 0.12
Vanilla bidi	0.66 ± 0.019	0.17 ± 0.003	0.11 ± 0.001	0.60 ± 0.001	20.20 ± 0.23

The data are reported as “mean ± 1 standard deviation” for triplicate measurements

**Table 5:** Concentrations (μg/bidi) of polar compounds for different size fractions of the particulate phase of bidi smoke as measured by GC–MS analysis

Compound	Filter		
	A (> 2.50 μm)	D (0.25–0.50 μm)	L (< 0.25 μm)
2-Methylphenol	0.65 ± 0.22	1.96 ± 0.17	13.26 ± 2.25
4-Methylphenol	3.35 ± 0.28	9.83 ± 3.14	15.93 ± 1.41
2-Methoxyphenol	1.20 ± 0.35	< DL^a^	45.33 ± 7.63
Indole	< DL	< DL	25.80 ± 4.94
Phytol	< DL	0.65 ± 0.06	20.86 ± 6.99
2,3-Dipyridyl	< DL	2.33 ± 0.21	46.74 ± 6.35
3-(3,4-dihydro-2*H*-pyrrol-5-yl)pyridine	0.67 ± 0.16	2.52 ± 0.67	52.08 ± 12.07
1*H*-Indole, 3-methyl	0.40 ± 0.01	0.38 ± 0.057	12.64 ± 1.075
Cotinine	0.30 ± 0.06	0.60 ± 0.12	32.60 ± 3.49
Vitamin E	< DL	0.74 ± 0.12	28.30 ± 4.83
Alpha-amyrin	2.30 ± 0.75	< DL	60.73 ± 4.49
Beta-amyrin	0.80 ± 0.22	< DL	56.36 ± 1.89

The data are reported as “mean ± 1 standard deviation” for triplicate measurements

^a^ < DL: Below detection limit

Table 6 shows the concentration ratio data of selected constituents in the gas phase as measured by the ASSET-32 sorbent tube analysis and the particulate phase of bidi smoke based on the Sioutas cascade impactor filter measurements along with their vapor pressure, aqueous solubility and Henry’s Law constant. Among non-polar volatile compounds, styrene and naphthalene have relatively high Henry’s constants of 2.82 × 10^−3^ atm m^3^/mol and 4.24 × 10^−4^ atm m^3^/mol, respectively. Compounds with higher aqueous solubility include 2-methylphenol and 4-methylphenol (31.8 g/L and 23.1 g/L, respectively).

**Table 6:** Physical properties and concentrations of selected bidi smoke components in vapor and particulate phases

Compound	Vapor pressure (mm Hg)	Solubility in water (g/L)	Henry’s constant (atm m^3^/mol)	Gas phase conc. (μg/piece)	TPM conc. (μg/piece)^a^	Ratio (gas/TPM)
2-Methylphenol	29.90 × 10^−2^	31.80	1.20 × 10^−6^	5.37	15.87	0.33
4-Methylphenol	11.00 × 10^−2^	23.10	1.00 × 10^−6^	15.10	29.11	0.51
2-Methoxyphenol	10.30 × 10^−2^	18.70	1.20 × 10^−6^	17.10	46.53	0.36
Cotinine	8.55 × 10^−5^	IS^b^	3.33 × 10^−12^	9.99	33.50	0.30

^a^TPM conc.: Total particulate matter concentration (sum of concentrations from filters A-L)

^b^IS: Insoluble

## Discussion

Many harmful and potential carcinogens were detected in the mainstream bidi smoke. High levels of carbon monoxide (Table 1), if inhaled, can enter into the bloodstream and bind with hemoglobin to form carboxyhemoglobin [5]. This results in tissue ischemia, and is a major cause of cardiovascular diseases, and is common among bidi smokers [9]. Hydrogen cyanide concentration in bidi smoke obtained from our studies is relatively low (310.8 µg/bidi) compared to an earlier report (688–904 µg/cigarette) [13]. The discrepancy in hydrogen cyanide concentrations can be attributed to the differences in the amount of tobacco per cigarette and the sampling conditions. The amount of nicotine in bidis is very high (21.1 mg nicotine/g tobacco) compared to the commercial filtered (16.3 mg nicotine/g tobacco) and unfiltered (13.5 mg nicotine/g tobacco) cigarettes [17]. Most previous studies have employed either a higher puff frequency or 15–60 s interval between puffs whereas, we performed our analysis with a 6 s inter-puff interval. In addition, the level of 1,3 butadiene is also high in bidi mainstream smoke (63.8 μg/bidi). Inhalation exposure to these compounds have been documented to have adverse health effects, as hydrogen cyanide is a ciliatoxin and 1, 3-butadiene is classified by Environmental Protection Agency (EPA) as a carcinogen [34, 35].

Due to the low combustibility of tendu leaves, bidi smokers tend to smoke with more intense puffs at higher puff frequency (4–6 puffs/min) than conventional cigarettes [17, 22, 36]. Therefore, the emission of different smoke constituents in a bidi was measured by sampling the smoke with increasing flowrate. As the flowrate increased from 0.56 to 1.25 L/min, the concentrations of carbon monoxide (CO), carbon dioxide (CO_2_), methanol (CH_3_OH), methane (CH_4_) ethane (C_2_H_6_), and ethylene (C_2_H_4_) decreased (Table 1). This general reduction in the concentrations of these compounds could be attributed to the dilution of combustion products with increased flowrate. The dilution effect is also related to the reduction in the collisional frequency of the intermediate radicals to form final combustion products, i.e. the combination of two methyl radicals lead to the formation of ethane. By taking the concentration ratios of each compound at the flowrate of 0.56 L/min relative to 1.54 L/min, an interesting descending trend of the concentration ratios in parentheses was observed for C_2_H_6_ (2.38), C_2_H_4_ (2.35), CO (2.00), CH_4_ (1.97), CO_2_ (1.88), and CH_3_OH (1.80). These data support two important observations. First, both the pyrolytic degradation of larger molecular weight compounds in bidis to yield C_2_H_6_ and C_2_H_4_ and the oxidative combustion processes to convert CO into CO_2_, and CH_4_ into CH_3_OH were important in determining the relative concentrations of hydrocarbons and oxidized products. Second, the oxidative combustion processes became progressively more prominent as indicated by the higher ratios of CO_2_/CO and CH_3_OH/CH_4_ as the flowrate was increased. This trend was consistent with the observed CO_2_/CO and CH_3_OH/CH_4_ concentration ratios of 13.13 (i.e. 30,340 ppm/2310 ppm) and 2.92 (i.e. 2860 ppm/980 ppm) at the flow rate of 1.54 L/min in comparison to the corresponding ratios of 12.30 and 2.66 at 0.56 L/min, respectively. The importance of flowrate in determining the relative proportions or concentrations of oxidized compounds compared to pyrolytically generated hydrocarbons is crucial in accurate measurements of the smokers’ exposure to specific harmful toxicants. Presently, there is no other study of the bidi smoke composition as a function of flowrate. Likewise, there has not been any systematic study of how the puffing duration and frequency would influence smoke composition. Future studies aimed at the use of more realistic smoking or puffing characteristics is necessary for determining the toxicant exposures among bidi smokers.

In the GC–MS analysis of ASSET-32 tube extracts with methanol and petroleum ether (Table 2), the match index for furfural in the petroleum ether extract was lower compared to that of the methanol extract (i.e. mass spectral match index of 76 versus 91 out of a perfect match index of 100) because of its chromatographic co-elution with 3-furaldehyde and 1,4-dimethypyrazole. The problem of co-elution for the extracts of bidi smoke is severe in the GC retention time range of 4–16 min. Therefore, in order to improve both the spectral match indices and quantitative accuracy, it is recommended that the quantitative analysis of bidi smoke be performed with extracted ion chromatograms for the specific and unique ions of the smoke toxicants.

Flavored bidis contribute an additional layer of complexity to the bidi smoke because of the flavor additives and their combustion products. Therefore, we investigated smoke constituents of three different flavored bidis. Analytes were sampled using ASSET-32 sorbent tubes and extracted with methanol for GC-MS analysis. Among other compounds such as nicotine, furfural and nicotyrine, bidi smoke had detectable amounts of 2-ethyl-3-hydroxy-4*H*-pyran-4-one which may have originated from the combustion of the tendu leaf. The pentacyclic triterpenes such as α-amyrin and β-amyrin, found in bidi smoke when analyzed from both methanol and petroleum ether extracts, have been shown to have insecticidal effects in plants. Detection of amyrin isomers in both methanol and petroleum ether extracts is in agreement with previous reports of amyrin being present in the cuticle of tendu leaves [18]. In the studies of mice treated with a mixture of α-amyrin and β-amyrin, both antihyperglycemic and hypolipidemic effects were observed, suggesting that it could be a drug candidate for treating diabetes and atherosclerosis [31]. The presence of substituted phenols such as cotinine, furfural and 2-furanmethanol (Table 3) can be harmful to human health. The relative toxicity of 253 substituted phenols found in mainstream cigarette smoke has been studied [32]. The quantitative structure activity relationship (QSAR) studies on phenols show that substituted phenols with electron-releasing groups can form potentially toxic phenoxyl free radicals whereas substituted phenols with electron-withdrawing groups exert their toxicity primarily through lipophilicity. The methyl and methoxy substituents of the phenols observed for bidi smoke are electron-donating and therefore capable of forming the phenoxyl free radicals. The relative standard deviation (RSD) values of compounds measured in bidi smoke were high due to the large variability in the amounts of tobacco and tendu leaf materials and their composition. This in turn is related to the fact that bidi manufacturing is a cottage industry that is highly unregulated with minimal automation and quality control [11]. Therefore, the higher RSD value or broader range of toxicant concentrations is also due to the variability associated with bidi tobacco processing and bidi rolling practices.

Presence of highest proportion of nicotine in Filter L of Sioutas cascade impactor (Table 4) suggests that most of the nicotine in the bidi smoke exists in the finest fraction of the particulate phase that reaches the alveolar tissue. The total nicotine found in the particulate phase of both vanilla and grape flavored bidi are higher compared to conventional cigarettes [17]. Since nicotine is a semi-volatile compound, it tends to partition into its equilibrium states in both the gaseous phase and the particulate phase. It can exist in either the protonated form (containing basic nitrogen atom) or in the non-protonated form (referred as the free base form) [25, 26]. In the gaseous phase, nicotine is primarily composed of the free base form because it is the only form that can volatilize [24]. On the other hand, since methylene chloride does not extract protonated or charged analytes in this study, nicotine detected in the TPM was of the non-protonated form.

Compounds detected in the particulate phase in different size filters are potential candidates to be deposited in different regions of respiratory tract (Table 5). Among them, compounds in the smaller particle size fractions make these compounds very harmful to human health because they can easily penetrate deep into tissues such as alveoli in the lungs. Since amines including nicotine, cotinine, myosmine, and indoles constitute approximately 60% of the particulate matter in Filter L and are highly soluble in blood, this enhances the rapid distribution of these potentially harmful compounds at relatively high concentrations to various tissues and organs of bidi smokers.

In order to understand the gas-particulate partition equilibria of bidi smoke constituents, the concentration ratio data of selected constituents in the gas phase as measured by the ASSET-32 sorbent tube analysis and the particulate phase of bidi smoke based on the Sioutas cascade impactor filter measurements were compared to the values of their vapor pressure, aqueous solubility and Henry’s Law constant (Table 6). The volatile and non-polar compounds generally have high gas-to-particulate concentration ratios because the higher vapor pressure or Henry’s constant of a volatile compound is associated with its predominant existence in the gas phase relative to the particulate phase, which is essentially viscous tar after agglomeration of the fine aerosol particles. Styrene and naphthalene, which have relatively high Henry’s constants (2.82 × 10^−3^ atm m^3^/mol and 4.24 × 10^−4^ atm m^3^/mol, respectively), are examples of non-polar volatile compounds that tend to exist at elevated levels in the gas phase instead of the aerosol phase. The low aqueous solubility of styrene and naphthalene also explain their presence in Filter L with the finest and hydrophobic particulate fraction of < 0.25 μm instead of Filters A-D because they do not partition readily into larger aqueous aerosol particles. On the contrary, polar compounds with the hydroxyl groups such as 2-methylphenol, 4-methylphenol and 2-methoxylphenolhave intermediate Henry’s constants of 1.0 × 10^−6^ atm m^3^/mol to 1.2 × 10^−6^ atm m^3^/mol and relatively higher aqueous solubility. This volatility trend implies that highly volatile and non-polar hydrocarbons exist almost exclusively in the gas phase while the phenols are found in both gas and particulate phases. The particulate phase of various size fractions have varying amounts of moisture resulting from the water produced by the bidi combustion. The formation of larger particles collected in Filter A results from the agglomeration of fine aerosol particles in the mainstream smoke. The smallest particulate fraction of less than 0.25 micron collected in the Filter L tends to be more hydrophobic and consists of non-volatile constituents such as cotinine, 2,3-dipyridyl, and α/β-amyrin, which have octanol–air partition coefficients of 0.145, 0.0192, and 0.812, respectively (http://www.chemspider.com/, [6]). These coefficients are higher than those of the three phenols with values on the order of 10^−5^–10^−6^, which explains why their mass percentages are higher than the phenols as shown in Table 5. In general, compounds with hydroxyl and amino functional groups including 2-methylphenol, 4-methylphenol, α/β-amyrin, nicotine, cotinine, and myosmine are commonly found in the particulate phase compared to small molecular weight and non-polar hydrocarbons that are predominantly in gas phase. These observations are consistent with the finding of a previous study stating that the gas-particle partition constant of nicotine decreases as humidity increases because the higher humidity promotes condensation to yield a greater amount of aerosol particles [15]. Thus, all the polar compounds are more likely to be readily absorbed and deposited into the respiratory tract of humans. Among other compounds detected in Filter L, while styrene, naphthalene, and 1,2-benzenediol may be potentially toxic [28], campesterol and stigmasterol are phytosterols from the tendu leaves that may impart beneficial cholesterol-lowering effects [16].

## Conclusion

Many chemical constituents detected in the mainstream bidi smoke are known toxins. For example, hydrogen cyanide is a ciliatoxin; both benzene and 1,3-butadiene has been classified as human carcinogens by EPA and International Agency for Research in Cancer (IARC) [34, 35]. Damage of respiratory tract cilia by hydrogen cyanide can severely impair mucociliary clearance in smokers and compromise their ability for clearing tar and other environmental pollutants in the respiratory tract. These pollutants deposit and then get absorbed in the body. In addition, exposures to high levels of these compounds are associated with eye irritation, giddiness, nausea, and may lead to cardiovascular and respiratory tract diseases [33, 34]. Nicotine, the major component of bidi smoke, was present at a significantly higher level in the particulate size fraction of < 0.25 μm. This implies that nicotine can be readily deposited and absorbed through human lung tissues. Delivery of these fine particles could occur via evaporative gas deposition (EGD) process in which nicotine vaporizes from the particulate phase into the gas phase and is subsequently deposited onto the human tissue [15]. The remaining nicotine in the TPM could get delivered into the respiratory tract via the particle deposition with evaporation (PDE) mechanism [24]. Here, the nicotine-containing particles first settle on the tissue before vaporization occurs that allows the subsequent deposition of nicotine from the gaseous phase for delivery. Another mechanism for nicotine delivery in the respiratory tract would be through the particle deposition with diffusion (PDD) process, where nicotine from the smoke deposits onto the tissue in particulate phase and then diffuses further into the tissue [24]. Among the compounds identified in bidi smoke, *o*-xylene or 1,2-dimethylbenezene is a known skin, eyes, and respiratory tract irritant. Due to its liposolubility, acute exposure of 1,2-dimethylbenezene may lead to skin inflammation, and toxicity of the respiratory tract and the central nervous system [2]. Health effects of many other compounds detected in this study are yet to be determined. Thus, bidi smoking raises concern about user awareness and safety. Unlike traditional cigarettes, where more than 6500 tobacco-specific chemicals have been identified [29] and some of them have detailed studies of their health impact, the research on bidi smoke is at its infancy. Therefore, more studies are required to investigate the complete chemical profile of bidi and evaluate their health impact.

## Data Availability

The datasets used and/or analyzed in this study are available from the corresponding author upon request.

## Abbreviations

ATSDR: Agency for Toxic Substances and Disease Registry
CDC: Centers for Disease Control and Prevention
EPA: Environmental Protection Agency
EGD: evaporative gas deposition
FTIR: Fourier-transform infrared spectrometry
GC–MS: gas chromatography coupled with mass spectrometry
IARC: International Agency for Research in Cancer
NIOSH: National Institute for Occupational Safety and Health
NIST: National Institute of Standards and Technology
PDD: particle deposition with diffusion
PDE: particle deposition with evaporation
PAHs: polycyclic aromatic hydrocarbons
QSAR: quantitative structure activity relationship
RSD: relative standard deviation
TSNAs: tobacco-specific nitrosamines
TPM: total particulate matter
VOCs: volatile organic compounds
WHO: World Health Organization
